# Editorial: The Roles of Mitochondria in Immunity

**DOI:** 10.3389/fimmu.2022.914639

**Published:** 2022-05-16

**Authors:** Naeem K. Patil, Julia K. Bohannon, Vidula Vachharajani, Charles E. McCall

**Affiliations:** ^1^ Department of Anesthesiology, Vanderbilt University Medical Center, Nashville, TN, United States; ^2^ Department of Pathology, Microbiology, and Immunology, Vanderbilt University Medical Center, Nashville, TN, United States; ^3^ Departments of Critical Care Medicine and Inflammation and Immunity, Cleveland, Clinic Lerner College of Medicine of Case Western Reserve University, Cleveland, OH, United States; ^4^ Department of Internal Medicine, Wake Forest School of Medicine, Winston Salem, NC, United States

**Keywords:** mitochondria, immunity, TCA cycle, itaconate, oxidative stress, inflammation

## Introduction

The manuscripts presented in our special topic - ‘The Roles of Mitochondria in Immunity’ enhance our understanding of the mechanistic link between mitochondrial function, tricarboxylic acid (TCA) cycle metabolites, inflammation, mitochondrial-derived reactive oxygen species (mROS), and mitochondrial quality control pathways in health and disease. Here, we highlight mitochondrial biology relevant to the special topic.

## Mitochondria Regulate Cellular Energy and Metabolism

As drivers of cell and organism fate and function, mitochondria generate most of the energy supply to meet the demands of dormancy, growth, replication, and survival. Under appreciated is the importance of mitochondrial energy network of the TCA cycle intermediates that promote diabetes, obesity and cancer (1, 2). Mitochondrial energy shortage underlies immunosuppressive pathologies and contributes to acute inflammatory syndromes of sepsis and immunosenescence. Cross–talk between the nuclear and mitochondrial genome regulates mitochondrial biogenesis, fusion, fission, and mitophagy (3), thereby controlling energy demand and supply. Mitochondria also are the predominant source of intracellular reactive oxygen species (mROS). Physiological levels of mROS are critical for cellular signaling pathways connected to nutrient metabolism and energy synthesis, Uncontrolled generation of mROS causes oxidative stress–induced cellular injury and death mechanisms (4, 5). TCA cycle balancing of anabolism and catabolism, as well as ATP levels relative to ADP and AMP play a critical role in cellular energy homeostasis.

## Inflammation Remodels TCA Cycle Citrate, α–Ketoglutarate, Succinate, Fumarate, Malate and Oxaloacetate

Inflammation reprograms mitochondrial metabolism leading to accumulation of key TCA cycle metabolites (6–9). Recent discoveries emphasize that mitochondrial metabolic remodeling during stress in innate immune cells and organ specific cells increases expression of the immune responsive gene 1 (Irg1) which redirects the TCA cycle by diverting citrate to itaconate (10). Other mitochondrial metabolites regulate diverse functions in leukocytes, including epigenetic modifications of inflammation, antimicrobial functions, and cell death or survival routes (11, 12). Citrate and succinate enhance leukocyte inflammation and immune resistance (13), while itaconate attenuates inflammation and promotes disease tolerance (14–16). Derivatives of itaconate, dimethyl itaconate analog (DMI) and 4–octyl itaconate (4OI) activate Nrf2, an antioxidant regulator and inhibit NLRP3 driven inflammation (15, 17). Some studies question the direct role of endogenous itaconate in repressing inflammation (18). The molecular biology of itaconate in non–immune cells is unclear.

## Knowledge About the Novel Anti–Inflammatory, Antioxidant, and Antimicrobial Effects of Itaconate and its Chemical Analogs Is Emerging

Oh et al. evaluated the effect of itaconate and its derivatives on muscle cell differentiation. Using an *in vitro* model of C2C12 muscle cells, this study supported that itaconate and its derivatives, DMI and 4OI, disrupt muscle cell differentiation by blunting the expression of key transcriptional and protein markers such as Myogenin (MYOG), which is required for myogenesis. Previous studies reported that itaconate regulates anti–inflammatory effects by limiting succinate dehydrogenase (SDH), at electron chain complex II (15, 19). Oh et al. showed in C2C12 cells that SDH inhibitors such as diethyl malonate, and exogenous succinate inhibit myogenesis, similarly to itaconate. 4OI also inhibits injury induced–MYOG expression *in vivo*. Thus, itaconate and its derivatives can interfere with myogenesis by inhibiting SDH function, highlighting itaconate signaling as a potential therapeutic target in muscle wasting states. Temporal assessments of itaconate on muscle physiology and mechanisms other than SDH inhibition are not reported, underscoring the need to identify non–immune and deleterious effects of itaconate in reducing inflammation, such as prolonged immunometabolic repression.

## mROS such as Superoxide and Excessive H_2_O_2_ Promote the Oxidant Stress of Sepsis–Induced Organ Injury, Cancer, Cardiovascular Diseases, and Numerous Inflammatory Conditions

Mitochondrial derived reactive oxygen species and oxidative stress contributes to numerous pathological conditions (20–23). Mizuguchi et al. investigated the role of complement component 1q subcomponent binding protein (C1qbp/p32), mitochondrial function and mROS in psoriasis. Mutations in C1qbp has been shown to cause defective mitochondrial function and cardiomyopathies (24). Using a hematopoietic cell–specific genetic deletion of C1qbp, Mizuguchi et al. demonstrated that C1qbp protects mice from imiquimod–induced psoriatic inflammation. Dendritic cells lacking C1qbp impair pro–inflammatory cytokine production including IL–1β, IL–23, and mROS upon imiquimod stimulation. Thus, mROS can promote psoriatic inflammation through C1qbp. Whether mROS should be investigated as a therapeutic target in psoriasis merits further research. The role of C1qbp–mROS in epithelial fate and function, and inflammasome biology in humans is unknown.

## Acetaminophen (APAP)–Induced Liver Injury Is A Critical Public Health Topic Due to its Prolific Use as an Analgesic and Antipyretic

APAP overdose can induce serious liver injury (25). Wang et al. investigated mROS–induced pyroptosis effects on APAP–induced liver injury and the countering effect of peroxiredoxin 3 (PRX3). PRX3 antioxidant mediator scavenges peroxide ROS. Pyroptosis is a form of inflammatory cell death caused by infection and the resulting inflammasome activation increases IL–1β and IL–18 proinflammatory cytokines (26). mROS activate hepatocyte pyroptosis controlled by the NLRP3 inflammasome, and APAP–induced liver injury increases oxidative stress and inflammation (26). Liver–specific silencing of PRX3 using short hairpin RNA (shRNA) technique augmented APAP–induced pyroptosis and liver injury, which was attenuated by the antioxidant, mito–TEMPO. Mitochondrial PRX3 limits mROS–induced oxidative stress and liver injury during APAP toxicity. In summary, PRX3 inhibits APAP–induced pyroptosis *via* inhibiting mitochondrial oxidative stress and NLRP3 activation.

## Impact of Oxalate and Taurine on Macrophage Metabolism and Antimicrobial Function

Patients with calcium oxalate (CaOx) kidney stones show repressed cellular energetics in peripheral monocytes (27). Healthy subjects consuming an oxalate enriched diet also show impaired monocyte mitochondrial function (28). Oxalate promotes pro–inflammatory macrophages (29). CaOx kidney stones increase susceptibility to urinary tract infections caused by uropathogenic *E. coli* (UPEC) (30). Tissue macrophages play a critical role in host antimicrobial defense, however the impact of oxalate on macrophage metabolism is not well defined. Using an *in vitro* model of human monocyte (THP–1 cells) derived macrophages, Kumar et al. demonstrated that oxalate treatment increased pro–inflammatory cytokine production (IL–6 and IL–1β), increased ROS levels, and reduced mitochondrial complex I and IV activities and ATP levels. Oxalate treatment also reduced macrophage antimicrobial capacity as demonstrated by reduced capacity to clear *E. coli*. These findings show that increased oxalate exposure increased oxidative stress and disrupted macrophage mitochondrial homeostasis and antimicrobial functions. Whether oxalate exposure also reprograms the TCA cycle leading to alterations of mitochondrial metabolites and inflammatory signaling pathways needs investigation.

Taurine plays a critical cytoprotective role, predominantly through augmenting cellular antioxidant defense and regulating other processes including autophagy, calcium homeostasis and cellular metabolism (31). Taurine supplementation decreases inflammation in chronic diseases such as diabetes and chronic obstructive pulmonary disease (COPD) (32, 33). However, the impact of taurine on regulating macrophage polarization during inflammation is not well defined. Meng et al. demonstrated that inflammation and high taurine exposure (LPS/IFNγ) upregulates the expression of taurine transporter (TauT/Slc6A6) during M1 macrophage polarization. Increased intracellular taurine levels impair methionine metabolism leading to low S–adenosylmethionine (SAM) levels, which is important for maintaining methylation of protein phosphatase 2A (PP2A). Methylation at the leucine–309 residue of catalytic C–subunit promotes macrophage–related inflammation in DSS–induced enteritis and endotoxin models (34, 35). Meng et al. further demonstrated that taurine inhibits PP2Ac methylation, thereby blocking PINK1–mediated mitophagy flux, as methylated PP2Ac served to activate PINK1. Taurine–exposed macrophages had reduced glycolytic capacity, higher pyruvate dehydrogenase activity, and increased ATP levels, thereby indicating a reversal of the Warburg effect typically observed in M1 macrophages. Inhibition of mitophagy, thereby blocking mitochondrial turnover, is the major mechanism responsible for taurine–induced macrophage metabolic reprogramming [Meng et al.]. These findings add to our understanding of macrophage phenotype in the context of taurine and mitochondrial quality control. Further studies using *in vivo* models of inflammation and primary human cells are warranted to confirm the translational relevance of these findings.

## Other Insights into Mitochondria and Immunity

Particulate matter (PM) exposure limits sperm quality and induces higher male infertility (36). A study by Zhu et al. demonstrated that PM exposure induced asymptomatic orchitis in mice by activating NF–κB signaling in testes. PM exposure reduced mitochondrial abundance and increased mitochondrial DNA release coupled with cGAS–STING inflammatory pathway activation. Treatment with aspirin–induced cGAS acetylation inhibited testicular inflammation in mice and rescued offspring growth in PM–expose mice [Zhu et al.]. Studies elucidating the exact molecular mechanisms leading to PM–induced orchitis and the role of mitochondrial dysfunction need defining.

Finally, Goldsmith et al. offer a perspective article highlighting recent studies exploring the links between exercise, on cellular metabolic pathways, and epigenetic reprogramming, particularly regarding T cell plasticity. Mitochondrial function, which can be greatly influenced by exercise, is critical in the generation of metabolites and cofactors needed for epigenetic reprogramming required for T cell stability. Among the effects of exercise on T cell plasticity include increased glycolysis, which regulates NAD+/NADH ratio (regulator of sirtuin histone deacetylase), limiting of glutamine metabolism, which reduces α–ketoglutarate levels (required for histone demethylase reactions), and increase in peripheral homocysteine levels, which alters the methionine: homocysteine ratio (regulator of DNA methylation reactions through one carbon metabolism and S–adenosylmethionine). Although more studies are needed to further identify the role of exercise on immune cell plasticity, these studies highlight the potential to modify mitochondrial function and epigenetic remodeling *via* exercise.

## Conclusion

In conclusion, 1.5–billion–year–old oxidative eukaryotic mitochondria primarily depend on pyruvate to direct the TCA cycle redox status and an energy supply chain that assures the universal principles of growth, replication, and survival. Acute or chronic disease occurs when mitochondrial control over the energy supply chain for homeostasis becomes inadequate or inflexible. This special edition in Frontiers Immunology enlightens the role of these critical organelles in immunity, health and disease.

## Author Contributions

All authors made a substantial intellectual contribution to the work and approved it for publication.

## Funding

CM is supported by NIH 5R35 GM126922, VV is supported by R01AA028763 (NIAAA) and R01GM099807 (NIGMS), JB is supported by NIH 5R35 GM141927, and NP was supported by Vanderbilt Faculty Research Scholars Award and the Shock Society Faculty Research Award.

## Conflict of Interest

The authors declare that the research was conducted in the absence of any commercial or financial relationships that could be construed as a potential conflict of interest.

## Publisher’s Note

All claims expressed in this article are solely those of the authors and do not necessarily represent those of their affiliated organizations, or those of the publisher, the editors and the reviewers. Any product that may be evaluated in this article, or claim that may be made by its manufacturer, is not guaranteed or endorsed by the publisher.
